# Mapping structural covariance networks in children and adolescents with post-traumatic stress disorder after earthquake

**DOI:** 10.3389/fpsyt.2022.923572

**Published:** 2022-09-15

**Authors:** Xian Mo, Meirong He, Lijun Zhou, Yunfei Liu, Hongru Zhu, Xiaoqi Huang, Guojun Zeng, Junran Zhang, Lingjiang Li

**Affiliations:** ^1^College of Electrical Engineering, Sichuan University, Chengdu, Sichuan, China; ^2^Med-X Center for Informatics, Sichuan University, Chengdu, Sichuan, China; ^3^Mental Health Center and Psychiatric Laboratory, West China Hospital, Sichuan University, Chengdu, Sichuan, China; ^4^West China Hospital, Sichuan University, Chengdu, Sichuan, China; ^5^Department of Psychiatry, The Second Xiangya Hospital, Central South University, Changsha, Hunan, China

**Keywords:** children and adolescents, post-traumatic stress disorder, cortical thickness, connectivity, magnetic resonance imaging

## Abstract

For children and adolescents, there is a high risk of developing post-traumatic stress disorder (PTSD) after suffering from catastrophic events. Previous studies have identified brain functionally and subcortical brain volumes structurally abnormalities in this population. However, up till now, researches exploring alterations of regional cortical thickness (CTh) and brain interregional structural covariance networks (SCNs) are scarce. In this cross-sectional study, CTh measures are derived from 3-Tesla Tl-weighted MRI imaging data in a well-characterized combined group of children and adolescents with PTSD after an earthquake (*N* = 35) and a traumatized healthy control group (*N* = 24). By using surface-based morphometry (SBM) techniques, the regional CTh analysis was conducted. To map interregional SCNs derived from CTh, twenty-five altered brain regions reported in the PTSD population were selected as seeds. Whole-brain SBM analysis discovered a significant thickness reduction in the left medial orbitofrontal cortex for the subjects with PTSD. Similarly, analysis of SCNs associated with “seed” regions primarily located in default mode network (DMN), midline cortex structures, motor cortex, auditory association cortex, limbic system, and visual cortex demonstrated that children and adolescents with PTSD are associated with altered structural covariance with six key regions. This study provides evidence for distinct CTh correlates of PTSD that are present across children and adolescents, suggesting that brain cortical abnormalities related to trauma exposure are present in this population, probably by driving specific symptom clusters associated with disrupted extinction recall mechanisms for fear, episodic memory network and visuospatial attention.

## Introduction

Posttraumatic stress disorder (PTSD) is a prevalent and disabling mental disorder characterized by a cluster of emotional and behavioral symptoms including re-experiencing, avoidance, hyperarousal, negative cognitions, and mood (1) related to the experience of catastrophic events. 20.52% of the youth population after suffering from injury developed PTSD (2), and the overall lifetime prevalence is 3–9% (3). Prior findings have suggested that children and adolescents are more vulnerable to developing PTSD after catastrophic events than are adults (4, 5). As a collective trauma caused by natural disasters, earthquakes have caused more extensive trauma to the public representing the negative impact on social processes at the collective level compared to individual crisis events (6). The devastating Wenchuan 8.0-magnitude earthquake resulted in heavy casualties and seriously affected approximately 46 million people, of whom a considerable number were suffering from PTSD (7). For minors, the psychological impact of the earthquake is extremely far-reaching. During the follow-up of 6, 12, and 18 months, the incidence of PTSD symptoms was 9.7%, 1.3%, and 1.6% respectively (8). And the prevalence of posttraumatic stress in adolescents until 8 years after the Wenchuan earthquake was still 1.9–2.7% (9). Moreover, there has been ample evidence indicating that juveniles display different pathogenesis of PTSD, in consequence of neurodevelopment (10), psychological tolerance (11), and individual differences (12). With the purpose of thwarting the negative impacts of mental trauma in children and adolescents, the emphasis of current research is on how to understand the neurobiological mechanisms associated with minors.

Nevertheless, most neuroimaging studies of PTSD have focused on adults. Only a few pieces of literature have reported brain imaging changes in children and adolescents after exposure to an injury (13, 14). Besides, the major findings in minors are regional functional alterations like the amygdala (15), medial prefrontal cortex (16), visual cortex (17), and anterior cingulate cortex (13). Understanding the brain structural changes in the children and adolescents after trauma who are developing and still malleable can provide a way to explore neuropathophysiological mechanisms of PTSD for countering negative consequences, particularly because some studies have found that even the mature brain can be for structural changes after treatment interventions in the adult population (18, 19). Structural neuroimaging research in pediatric posttraumatic patients can potentially help illuminate the relationship between PTSD and brain structure, however, up till this moment, such studies are scant (14).

Cortical thickness (CTh) is a relatively novel structural neuroimaging analysis technique estimated generally by surface-based method, which can reflect changes in the cerebral cortex with the normal aging process and various nervous system diseases (20). Compared to the voxel-based morphometry (VBM) method, CTh determined by surface-based morphometry (SBM) might detect the delicate abnormities of brain structure more acutely (21, 22). However, most of the brain structure imaging studies of PTSD is based on the VBM method, among which adult patients are mostly focused. And the structural brain changes in adult patients with PTSD reported are mainly in the volumes of the hippocampus, amygdala, insula, medial prefrontal cortex, anterior cingulate, etc., (23–25). So far relevant pieces of research on structural neuroimaging analysis in children and adolescents with PTSD are still limited and no consistent conclusion has been drawn, particularly on cortical thickness. To our knowledge, there are only three reports on CTh about PTSD patients up to now, not including minors having experienced earthquakes (26–28). Ahmed et al. (26) found subjects with PTSD indicated a marked reduction in the insula thickness by using Freesurfer analysis on Qdec. A different result is reported by Rinne-Albers et al. (27) who found there was no significant difference in CTh between pediatric posttraumatic patients and controls in four selected ROIs including the insula. Moreover, the method of brain structural covariance networks (SCNs) constructed from inter-regional correlations estimated according to a group of individual images is a relatively untapped resource to be applied to reveal inter-regional co-variance patterns across the population of brain disorders like early psychosis (29–31). Importantly, for children and adolescents, SCNs as the consequence of interaction and promotion during brain development and maturation could help further understand the abnormal alteration of the morphometry relationships between different parts of the developing brain (30). In summary, a combined method involving CTh and SCN analysis may provide underlying information on the regional structure and interregional network relationships related to pediatric posttraumatic patients, which has not been reported in the current literature yet.

In this cross-sectional neuroimaging study, structural and network-level substrates of PTSD in children and adolescents after the same earthquake were assessed by using CTh analysis. Here, we hypothesized that altered CTh and covariance strength derived from 3-Tesla MRI in pediatric posttraumatic patients compared to traumatized non-PTSD subjects. First, regional cortical thickness analyses between groups were conducted to test for regional hypotheses. Next, we collected the mainly abnormal brain regions originally identified in the published neuroimaging meta-analyses on PTSD and restrained them to these which are reported at least in two individual meta-analyses. Furthermore, for measuring the correlation strength of CTh between these synthesized brain regions and all other brain vertices, a seed-based SCN analysis was used on the interregional network-level.

## Materials and methods

### Participants

Fifty-nine right-handed subjects who all experienced the same Wenchuan 8.0-magnitude earthquake were recruited (Table 1). The acquisition of neuroimaging and clinical data from survivors took place in December 2009, 17 months after this disaster. All participants were children or adolescents, who were between 8 and 18 years of age (35 PTSD with a mean age of 14.74 ± 2.08 years and 24 non-PTSD controls with a mean age of 14.58 ± 1.79 years) and didn’t suffer any physical head injury or any loss of consciousness > 5 min in the catastrophe. They were drawn from a large-scale PTSD survey of survivors 8–15 months after the earthquake. They were first carefully screened through the PTSD checklist (PCL) (32, 33). Subjects with a PCL score > 35 further participated in interviews led by two experienced psychiatrists. The Clinician-Administered PTSD Scale (CAPS) (34) was used to confirm the PTSD diagnosis in these individuals with suspected PTSD, and the structured clinical interview for DSM-IV (SCID) was used to exclude any psychiatric comorbidities. Those who scored > 50 on the CAPS were eligible for further evaluation for inclusion in the PTSD group. An age-and sex-matched trauma-exposed non-PTSD group was formed from those with a PCL score < 30 points and a CAPS score < 35 points. The exclusion criteria for the PTSD group were: (1) history of other nervous system diseases and psychiatric disorders; (2) use of psychotropic medications and drug treatment in the past 2 months; (3) any significant medical or history of head injury; (4) left-handedness; (5) IQ < 80; (6) magnetic resonance imaging contraindications. The traumatized control participants were similarly screened using the SCID and CAPS scale. These subjects who experienced the same traumatic events but with CAPS scores below 35 were included in the non-PTSD group. The non-PTSD control group used the same exclusion criteria as the PTSD group. An experienced neuroradiologist inspected brain scans and ruled out clinical abnormalities. This research was approved by the Medical Ethics Committee of West China Hospital, Sichuan University, and all subjects and their guardians offered written informed consent after being provided a complete description of this study.

**TABLE 1:** Sample characteristics.^a^

Clinical information	PTSD (*N* = 35)	Non-PTSD (*N* = 24)	Test statistics^b^
Age (years)	14.74 ± 2.08	14.58 ± 1.79	0.592
Gender (female/male)	23/12	14/10	0.565
Education (years)	8.43 ± 1.99	8.54 ± 1.64	0.509
CAPS (total)	7.01 ± 5.78	67.90 ± 15.23	< 0.001
Handedness (right/left)	35/0	24/0	–
Height (cm)	157.89 ± 8.92	158.50 ± 8.80	0.934
Weight (kg)	51.25 ± 8.01	49.95 ± 9.55	0.430

^a^Presentation of characteristics is mean ± SD. ^b^Two-sample *t*-test was used to test continuous characteristics and categorical characteristics were tested by chi-square.

### Magnetic resonance imaging acquisition

Images were acquired on a Siemens 3.0T Trio TIM MRI scanner at the West China Hospital. All subjects underwent high resolution three-dimensional T1-weighted anatomical brain scans (MPRAGE, 176 slices, TR/TE = 1900/2.26°msec, flip angle = 9°, acquisition matrix = 256 × 256, resolution = 1 × 1 × 1°mm^3^).

### Measurement of cortical thickness

The T1-weighted MRI data were preprocessed in FreeSurfer (Version 7.1.0^1^). The image processing includes the following main steps: motion correction, skull strip, Talairach transform computation, subcortical grey matter (GM)/white matter (EM) segmentation, intensity normalization, WM-GM boundary tessellation, automatic topology fixer, and spherical registration. Then, the cortical surface of individuals is registered to a standard spherical map after inflation by the spherical registration method. And sulcal and gyral features were recognized automatically by the software. The CTh data were smoothed by a 10-mm FWHM Gaussian kernel for reducing measurement noise and improving statistical power.

### Region-of-interest definition

We predefined twenty-five region-of-interests (ROIs) according to our previous research (35) in Table 2. The specific strategy was followed: each candidate seed was originally identified in at least two individual published neuroimaging meta-analyses on PTSD. These ROIs were modeled by spheres of 20°mm diameter around each peak coordinate and chosen as seeds to create structural covariance connectivity maps for a hypothesis-driven approach.

**TABLE 2:** Coordinates-region-of-interests (ROIs).

Number	Brain areas	Left/Right (L/R)	Peak coordinates		
			X	Y	Z
1	hippocampus	L	−28	−12	−14
2	amygdala	L	−20	−8	−12
3		R	24	−2	−16
4	medial prefrontal cortex/anterior cingulate cortex	L	0	20	26
5		R	12	36	18
6	insula	L	−42	8	−4
7		R	42	2	12
8	medial frontal gyrus	L	−34	4	52
9		R	12	42	24
10	caudate/putamen	L	−8	2	14
11	inferior frontal gyrus	R	46	16	10
12	precuneus	L	−10	−52	42
13		R	10	−52	46
14	posterior cingulate cortex	L	−2	−42	20
15	fusiform gyrus	L	−46	−42	−12
16		R	36	−72	−10
17	superior temporal gyrus/angularis	L	−50	−2	2
18	mid-cingulate cortex	L	−2	−16	36
19		R	8	16	34
20	inferior parietal lobule	R	32	−50	48
21	middle occipital gyrus	L	−32	−86	0
22	superior frontal gyrus	L	−24	52	10
23	precentral gyrus	L	−36	−8	38
24		R	40	10	40
25	inferior temporal gyrus	L	−48	−10	−20

### Statistical analyses

All cortical statistical analyses were performed using the SurfStat toolbox^2^ within MATLAB www.mathworks.com. And for the problem of multiple comparisons, each cluster was corrected with a random-field-theory (RFT) (36) at a*p* < 0.05 level of significance, limiting the probability of reporting a family-wise error to below 0.05. Two-sample *t*-test and chi-square analyses in SPSS software^3^ were used to compare the demographic characteristics between trauma-exposed non-PTSD and PTSD participants.

#### Regional substrates: Cortical thickness analysis between groups

In order to obtain the cortical thickness *T_i* of each cortical surface point *i*, general linear models (GLM) were used for fitting calculation:

$$T_{i}\,i\,n\,t\,e\,r\,c\,e\,p\,t+\beta_{1}\,\left(G\,r\,o\,u\,p\right)+\beta_{2}\,\left(A\,g\,e\right)+\beta_{3}\,\left(G\,e\,n\,d\,e\,r\right)$$

In the formula above, β_1_ values represent variable of interest and other β_2−3_ represent covariates, considering the notable impacts of Age and Gender on cerebral cortex (37).

#### Interregional network substrates: Structural covariance networks analysis

To map interregional SCNs, the CTh of each ROI was correlated with the thickness measurements across all cortical surface points. For each seed, we fitted interaction models that included terms for seed thickness, group, and their parametric interaction to assess group differences in seed-based structural covariance. The linear model at the cortical surface point *i* was fitted to calculate:

$$T_{i}∼i\,n\,t\,e\,r\,c\,e\,p\,t+\beta \,1\,\left(G\,r\,o\,u\,p\right)+\beta \,2\,\left(S\,e\,e\,d\right)+\beta \,2\,\left(A\,g\,e\right)+\beta \,4\,\left(G\,e\,n\,d\,e\,r\right)+\beta \,5\,\left(G\,r\,o\,u\,p^{*}\,S\,e\,e\,d\right)$$

where * denotes an interaction. As before, we regressed the covariates of age and gender.

## Results

### Samples characteristics

The comparisons of demographic characteristics between trauma-exposed non-PTSD and PTSD participants are provided in Table 1. The average gender and age of PTSD individuals were alike to the control’s, as were education and height, and weight. In addition, the groups differed in CAPS scores.

### Regional substrates: Cortical thickness analysis

**FIGURE 1:**
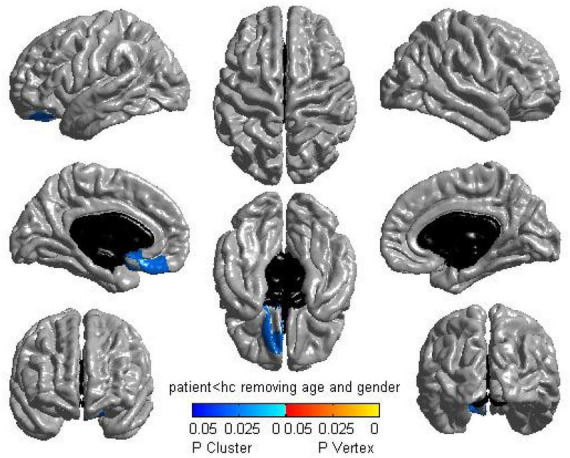
Regional cortical thickness analysis between groups. The surface-based morphometry analysis highlighted a significant thickness reduction in the left medial orbitofrontal cortex for post-traumatic stress disorder (PTSD) participants compared with trauma-exposed non-PTSD subjects [1,994 vertices, *P* = 0.0265, corrected with random-field-theory (RFT)].

The Surface-based morphometry analysis of CTh highlighted that PTSD patients displayed a significantly thinner cluster of the left medial orbitofrontal cortex (mOFC) than non-PTSD control subjects (*P* = 0.0265, corrected with RFT) (Figure 1). No difference was seen in PTSD patients> non-PTSD subjects.

### Interregional network substrates: Structural covariance networks analysis

**FIGURE 2:**
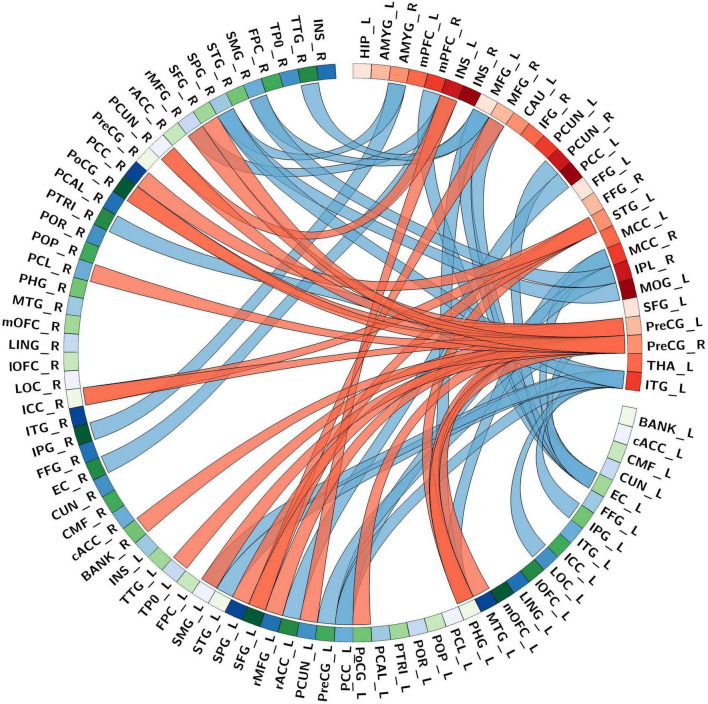
The structural connectivity network profile between the abnormal seed areas and the whole-brain cortical surface points in a combined sample of post-traumatic stress disorder (PTSD) group and non-PTSD. Bands with red and blue represent increased and decreased covariance, respectively. Ring color with gradient red and green-blue represents areas in the collected abnormal seeds and the whole-brain cortical surface, respectively. And the Desikan-Killiany atlas (thirty-four regions/per hemisphere) underlay the whole-brain cortical parcellations. As for abbreviations on the drawing, please refer to Supplementary Table 1.

The seed-based structural connectivity analyses between PTSD and non-PTSD control groups are presented in Figure 2 and Table 3. Findings showed that there were three types of alteration of cortical structural covariation for seed regions of interests: positive contrast (PTSD groups > non-PTSD controls), negative contrast (PTSD groups < non-PTSD controls), and both positive and negative contrasts. When considering positive contrast, there were two seed regions located in the auditory association cortex [left superior temporal gyrus (STG)] and motor cortex (right precentral gyrus) that showed increased covariance with other brain vertices in PTSD patients compared with non-PTSD participants between CTh. When considering the negative contrast, the results showed that lesser connectivities of ten seed regions located in the default mode network (DMN) regions [including left posterior cingulated cortex (PCC), right precuneus, right medial prefrontal cortex/anterior cingulate cortex (mPFC/ACC) and right inferior parietal lobule (IPL)], midline cortex structures [including bilateral medial frontal gyrus (MFG), right mid-cingulate cortex (MCC)], visual cortex [including left middle occipital gyrus (MOG) and left inferior temporal gyrus (ITG)], and limbic system (right amygdala) were found in PTSD patients compared with non-PTSD participants between CTh. When both positive and negative sides were considered, there were two seed regions including the left insula and precentral gyrus.

**TABLE 3:** Abnormal seed-based structural connectivity between post-traumatic stress disorder (PTSD) and non-PTSD (cluster *p* < 0.05, p-random-field-theory (RFT) corrected).

Seed numbers and regions	Peak of clusters			NVtxs	p-RFT	Anatomical location of clusters
	X	Y	Z			
**Positive contrast (PTSD > non-PTSD)**						
17 L superior temporal gyrus	−9	16	39	2986	0.002	L superior frontal
	−23	−22	−18	38	0.006	L parahippocampal
	4	−48	33	2204	0.01	R posterior cingulate R isthmus cingulate
24 R precentral gyrus	40	−26	56	4605	0.0003	R postcentral
	24	8	58	3010	0.002	R superior frontal
	−58	−25	31	3500	0.004	L supramarginal
	−21	−18	−22	35	0.0089	L parahippocampal
	47	−40	1	2334	0.03	R bankssts
	15	−31	39	1731	0.049	R paracentral
**Negative contrast (PTSD < non-PTSD)**						
3 R amygdala	35	−46	38	3934	0.0005	R superior parietal R inferior parietal
5 R medial prefrontal cortex	−21	1	−25	111	0.00003	L entorhinal
9 R medial frontal gyrus	−24	−1	−23	111	0.00004	L entorhinal
13 R precuneus	−23	3	−25	206	0.0004	L entorhinal
14 L posterior cingulate cortex	−2	29	−4	74	0.02	Lrostral anterior cingulate
19 R mid-cingulate cortex	−19	1	−30	112	0.0001	L entorhinal
	−28	−14	−35	1548	0.00999	L parahippocampal L entorhinal
20 R inferior parietal lobule	−2	−18	29	51	0.0068	L posterior cingulate
	64	−42	23	2351	0.015	R supramarginal
21 L middle occipital gyrus	30	−51	45	2169	0.02	R superior parietal
25 L inferior temporal gyrus	54	30	−2	2317	0.003	R pars triangularis
	33	−47	40	2775	0.004	R superior parietal
	57	−33	17	2836	0.01	L supramarginal L superior temporal
	−46	−68	9	1952	0.01	L lateral occipital L inferior parietal
	−3	−28	28	48	0.049	L posterior cingulate
8 L middle frontal gyrus	−19	2	−30	2178	0.00002	L parahippocampal L entorhinal
	41	−30	13	2894	0.02	R supramarginal R transverse temporal
**Two-sided contrast (both positive and negative)**						
6 L insula (positive)	−5	39	52	2780	0.0037	L superior frontal
	38	30	42	2195	0.0083	R rostral middle frontal
	−9	−56	64	2098	0.02	L precuneus
	8	−54	26	1846	0.04	R precuneus
6 L insula (negative)	24	−6	−29	40	0.0097	R entorhinal
23 L precentral gyrus (positive)	54	−11	33	9343	0.00001	R postcentral
	29	42	32	3613	0.0003	R rostral middle frontal
	−27	−39	53	3754	0.003	L postcentral L superior parietal
	−16	−66	64	2701	0.003	L middle temporal L temporal pole
	−26	55	17	2387	0.007	L rostral middle frontal
				2556	0.009	R precuneus R isthmus cingulate
	18	−55	5	1914	0.02	L superior parietal
	−22	−20	−20	28	0.03	L parahippocampal
23 L precentral gyrus (negative)	42	6	25	5375	0.0003	R precentral
	−27	−2	−22	54	0.004	L entorhinal

NVtxs, number of vertices; L, left; R, right.

**FIGURE 3:**
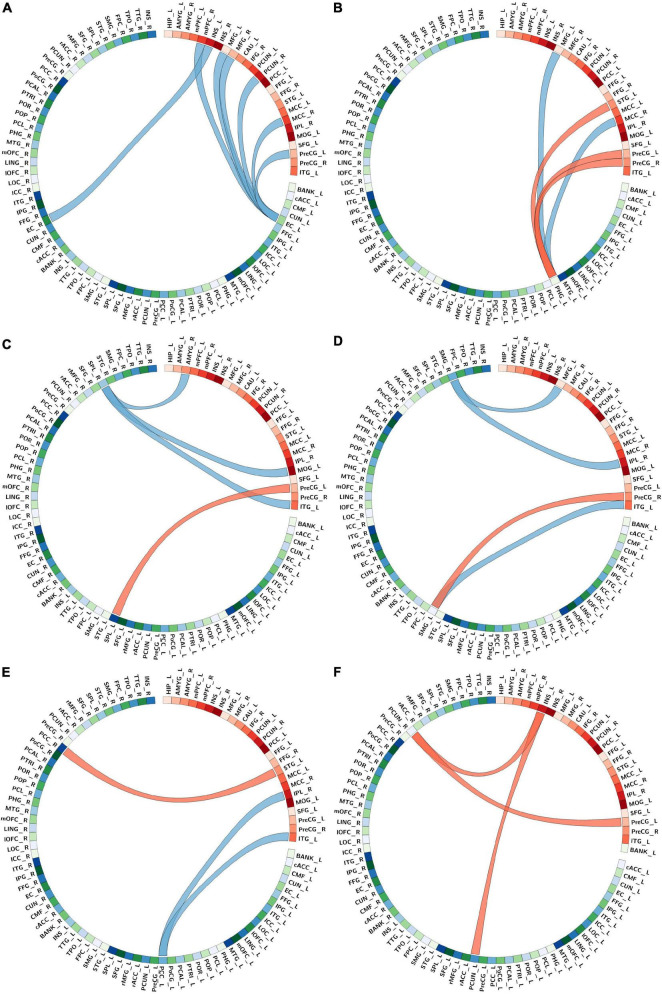
The main altered connectivity network profiles in structural covariance between post-traumatic stress disorder (PTSD) and none-PTSD group, centered on six key regions. **(A)** The connectivity network profiles of the right medial prefrontal cortex (mPFC), right precuneus, right mid-cingulate cortex (MCC), left insula, left precentral gyrus, and bilateral medial frontal gyrus (MFG) with the bilateral entorhinal. Bands with red and blue represent increased and decreased covariance, respectively. Ring color with gradient red and green-blue represents areas in the collected abnormal seeds and the whole-brain cortical surface, respectively. And the Desikan-Killiany atlas (thirty-four regions/per hemisphere) underlay the whole-brain cortical parcellations; **(B)** the connectivity network profiles of the left MFG, left superior temporal gyrus (STG), right MCC, and bilateral precentral gyrus with the left parahippocampal; **(C)** the connectivity network profiles of the right amygdala, left middle occipital gyrus (MOG), left precentral gyrus, and left inferior temporal gyrus (ITG) with bilateral superior parietal gyrus; **(D)** the connectivity network profiles of the left MFG, right inferior parietal lobule (IPL), right precentral gyrus, and left ITG with bilateral supramarginal gyrus; **(E)** the connectivity network profiles of the left STG, right IPL, and left ITG with the bilateral posterior cingulated cortex; **(F)** the connectivity network profiles of the left insula and precentral gyrus with the bilateral precuneus bilateral. As for abbreviations on the drawing, please refer to Supplementary Table 1.

In summary, covariance networks found were centered on six key regions, and representative images are shown in Figure 3. These were connectivities: (1) of seed regions including the right mPFC/ACC, right precuneus, right MCC, left insula, left precentral gyrus, and bilateral MFG with the bilateral entorhinal (Figure 3A); (2) of seed regions including the left MFG, left STG, right MCC, and bilateral precentral gyrus with the left parahippocampal (Figure 3B); (3) of seed regions including the right amygdala, left MOG, left precentral gyrus, and left ITG with the bilateral superior parietal gyrus (SPG) (Figure 3C); (4) of seed regions including the left MFG, right IPL, right precentral gyrus, and left ITG with the bilateral supramarginal gyrus (SMG) (Figure 3D); (5) of seed regions including the left STG, right IPL, and left ITG with the bilateral PCC (Figure 3E); (6) of seed regions including the left insula and precentral gyrus with the bilateral precuneus (Figure 3F). All these findings survived the p-RFT < 0.05 correction. For RFT-corrected maps depicting SCNs of all reported findings, please see Supplementary Figures 1–18.

## Discussion

The current research provides insights into the regional structure and interregional network differences of posttraumatic patients, in a well-characterized combined sample of children and adolescents after an earthquake, between 8 and 18°years of age. By using surface-based morphometry analysis, we analyzed CTh and examined the structural covariance connectivity of the abnormal regions involved in PTSD on network-level between participants with and without PTSD. As expected, on the structural whole-cortex vertex-wise level, a significant reduction in CTh was detected in the left mOFC. In the interregional structural covariance analysis, several seed regions mainly located in the DMN regions, midline cortex structures, motor cortex, auditory association cortex, limbic system, and visual cortex showed altered structural connectivities with six key regions: the bilateral entorhinal; the left parahippocampal; the bilateral SPG; the bilateral SMG; the bilateral PCC and the bilateral precuneus compared the PTSD subjects with the non-PTSD.

### Alterations in regional cortical thickness between groups

Post-traumatic stress disorder (PTSD) patients highlighted significantly thinner cortex in the left mOFC than non-PTSD groups. One of the leading factors in PTSD is the failure of fear extinction, and the reported mOFC which plays an important role in extinction learning is closely associated with the fear loop (38–40). The mOFC in classical experiments (41–43) has been widely considered to be involved in the retention or recall of extinction learning. In addition, the brain activity in the mOFC region decreased during experiencing a trauma (44), which seemed to reflect the lack of the “switch-off” mechanism of fear at this point (45). Furthermore, evidence from neuroimaging research suggested a positive correlation between the cortex thickness of mOFC and extinction recall (46, 47). And Morey et al. found that there were smaller orbitofrontal volumes in minors with PTSD (48). Taken together, individuals will maintain a better extinction ability to respond to fear when mOFC regions are activated more. In contrast to that, the thinner cortex in the mOFC of PTSD patients found in this research may reflect the reduced flexibility to control fear and may make children and adolescents more vulnerable to traumatic events. Therefore, the abnormalities in mOFC areas might be the outcome of disrupted extinction recall mechanisms for fear in PTSD.

### Alterations in interregional structural covariance networks

Covariance networks in the current study were significantly correlated with brain activations in areas centered on six key regions: the bilateral entorhinal, the left parahippocampal, the bilateral SPG, the bilateral SMG, the bilateral PCC, and the bilateral precuneus. Previous studies on the SCNs of PTSD have shown the structural integrity of the cingulate region, the cingulum bundle, and/or the amygdala or amygdala and other frontal regions in the adult population (49–52). At the same time, there are only a few reports about the structural connectivity changes derived from cortical thickness for minors with PTSD, which reported abnormalities in the left PCC, the right inferior frontal cortex, and the left ACC (53, 54). The current study discovered abnormal integrations of the structural connectivity of the entorhinal cortex, parahippocampal, and parietal cortex for children and adolescents with PTSD. These extensive abnormalities of integrated changes of structural connectivity may reveal why children and adolescents might be more vulnerable to the harmful effects of disastrous events, which will continuously threaten the health and welfare of current and future generations calling for more post-disaster policy attentions to the trauma problems faced by children in the process of growth and development after the earthquake and giving them long-term interventions and support (55, 56).

The entorhinal, parahippocampal, PCC, and precuneus are important components of the episodic memory network which have been proved to be often activated during episodic recall in PTSD patients by imaging studies (57–62). The entorhinal cortex and parahippocampal work together for spatial positioning and the formation of declarative memory (63). As the primary interface for information flow between the neocortex and the parahippocampal formation, the entorhinal cortex receives multimodal information from other cortical regions and then transmits them to the parahippocampal (64). We observed reduced structural connectivities in DMN regions (including right mPFC and right precuneus), midline cortex structures (including bilateral MFG and right MCC), the motor cortex (right precentral gyrus) with the left entorhinal and in left insula with the right entorhinal, which may be a result of inhibition of information flow between entorhinal cortex and neocortex associated with unique symptoms of emotional processing, re-experiencing memory and awareness of bodily states in PTSD youths. Additionally, the disturbance of structural connectivity in the parahippocampal might be the basis for sensory and contextual memory defects in posttraumatic patients (65, 66). The results that there were decreased structural connectivities in the midline cortex structures (including left MFG and right MCC)) and increased structural connectivities in the motor cortex (right precentral gyrus) and auditory association cortex (left STG) with the left parahippocampal gyrus supported the dual representation theory of PTSD (67), which suggest a dissociation between sensory and contextual memory representations in PTSD. This abnormal alteration of connectivity in the left parahippocampal gyrus is consistent with the previous structural covariance network studies showing that there was an increased correlation between the limbic system and the visual-related cortex (51) and impaired integration of the prefrontal limbic network with other parts of the brain in PTSD patients (68). Similar dissociation between hyperactive sensorimotor regions and hypoactive memory-associated regions was reported during PTSD-related flashbacks (69, 70). Viard et al. indicated decreased within-DMN connectivity of PCC in adolescents with PTSD (71), so the decreased structural connectivity in the visual cortex (left ITG) and DMN regions (right IPL) is known for their roles in visual mental imagery and contextual cue processing (67, 72) with the left PCC and the increased structural connectivity between auditory association cortex (left STG) and the right PCC might relate with distorted images and sounds, dysfunctional autobiographical memory retrieval. The precuneus which responds to multiple cognitive processes could be divided into regions involved in cognition, sensorimotor, and visual processing (73). Our results that seed regions in the left insula and precentral gyrus were structurally connected with the bilateral precuneus confirm this separation and might help explain the complex clinical manifestations of PTSD patients. From the above, our findings of altered structural connectivity of the four areas in the episodic memory network may be the foundation of trauma re-experiencing and recalling in children and adolescents with PTSD.

In addition, there were different connections in the bilateral SPG and SMG for PTSD patients compared with controls. The SPG is known for a crucial role in the early integration of visuospatial information carried by somatosensory, proprioceptive, and auditory stimuli (74, 75). And SMG is the secondary somatosensory cortex which can integrate exogenous and internal-sensory information (76). The precentral gyrus has an increased structural connection with SPG and SMG may indicate that PTSD patients are more difficult to process tactile and proprioceptive visuospatial sensory information related to external cues to carry out somatic drive. The decreased connection in the amygdala, MOG, and ITG with SPG suggests that PTSD patients have a reduced ability to integrate visuospatial information. Besides, the decreased connection between the inferior temporal gyrus and supramarginal gyrus may reveal the decreased spatial perception of PTSD patients. Consequently, these connection changes in SPG and SMG may reflect the reduction of visuospatial information integration ability in the youth population with PTSD.

## Conclusion

This research supplemented deeper insights to structure and network substrates of posttraumatic patients in children and adolescents, suggesting that cortical alterations related to trauma exposure are present in this population, probably by driving specific symptom clusters associated with disrupted extinction recall mechanisms for fear, episodic memory network, and visuospatial attention. In other words, connections in certain cortical regions may underlie the presentation of certain symptoms. These changes in regional and interregional structural features have significant implications for understanding the neural underpinnings of young posttraumatic patients.

However, several limitations deserve attention. Firstly, due to the limited sample size of subjects, regional alterations between groups may lack sensitivity and results should be considered rather preliminary. Secondly, for the control group, only traumatized non-PTSD individuals were included but healthy controls who had not experienced a traumatic event were not. Finally, current interregional analyses are conducted at the group-level, only reflecting differences in co-variance networks between two populations not including modulation effects of SCN by clinical characteristics. In the future, further individualized structural covariance networks analysis will be implemented using a larger sample of posttraumatic patients and adding non-traumatized healthy controls to explore individual differences among children and adolescents and modulation effects of cortical thickness by clinical characteristics.

## Data availability statement

The original contributions presented in this study are included in the article/Supplementary material, further inquiries can be directed to the corresponding authors.

## Ethics statement

The studies involving human participants were reviewed and approved by the Medical Ethics Committee of West China Hospital, Sichuan University. Written informed consent to participate in this study was provided by the participants’ legal guardian/next of kin.

## Author contributions

XM and JZ designed the study. LL, GZ, XH, and HZ organized the database. XM performed the statistical analysis and wrote the first draft of the manuscript. MH, LZ, YL, and XM wrote sections of the manuscript. All authors contributed to the article and approved the submitted version.

## Footnotes

1
http://surfer.nmr.mgh.harvard.edu

2
https://www.math.mcgill.ca/keith/surfstat/

3
https://www.ibm.com/analytics/spss-statistics-software
