# Supplementary material for: Mapping structural covariance networks in children and adolescents with post-traumatic stress disorder after earthquake
Source: Front Psychiatry. 2022 Sep 15;13:923572. doi: 10.3389/fpsyt.2022.923572 (PMC9520616; doi:10.3389/fpsyt.2022.923572)
Supplement: Supplementary file 1 [file Image_1.PDF]

## *Supplementary Materials*

**SUPPLEMENTARY FIGURE 1**

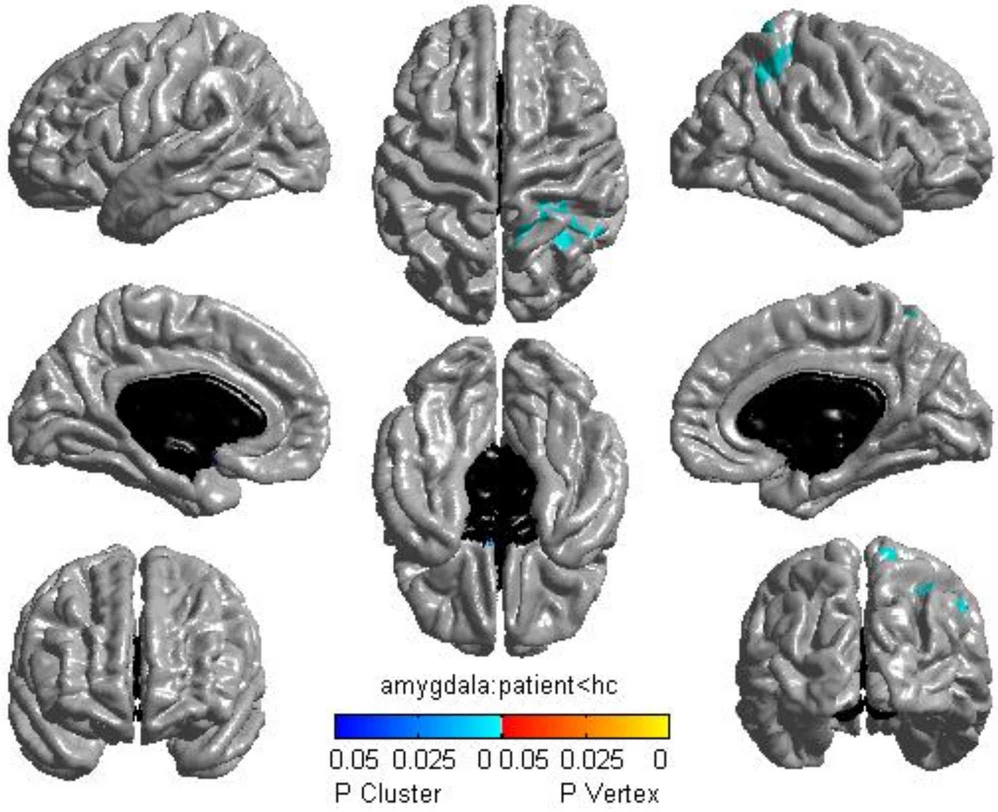


**SUPPLEMENTARY FIGURE 1** RFT-corrected map depicting seed-based structural covariance networks of the right amygdala seed region. Reduced covariance was found overall in PTSD groups compared with non-PTSD controls between cortical thickness in the right amygdala seed region of interest and cortical thickness in the right superior parietal gyrus and inferior parietal gyrus (3934 vertices, p=0.0005, corrected with RFT).

**SUPPLEMENTARY FIGURE 2**

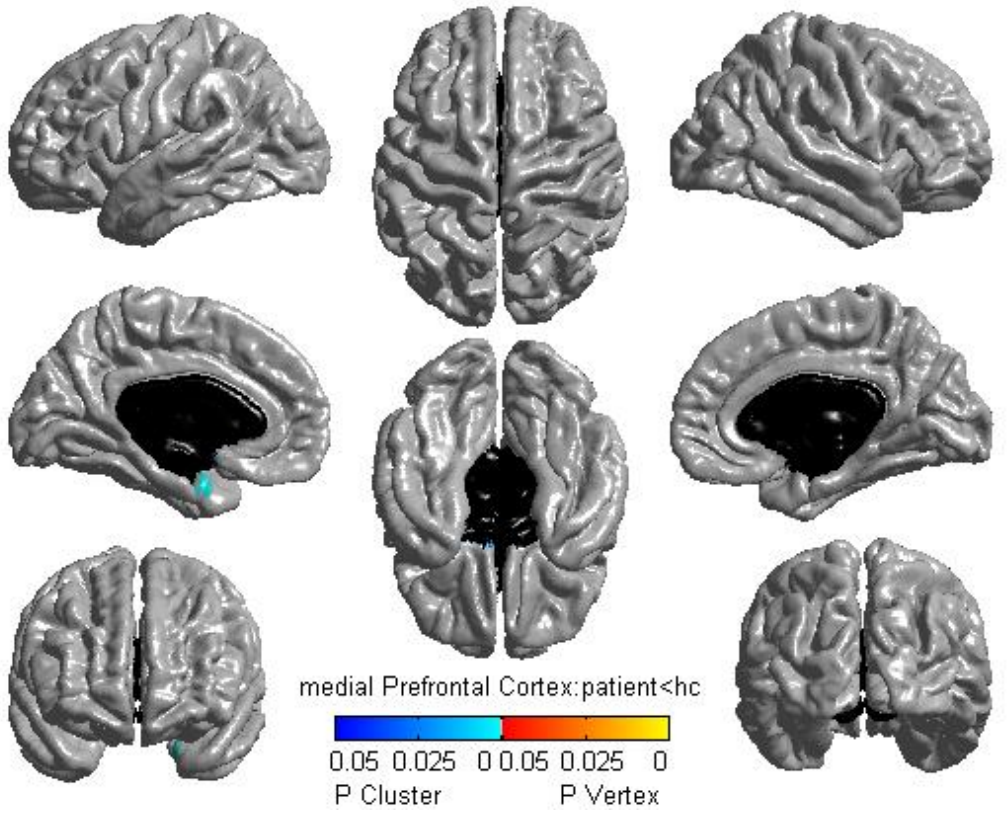


**SUPPLEMENTARY FIGURE 2** RFT-corrected map depicting seed-based structural covariance networks of the right medial Prefrontal Cortex/ Anterior Cingulate Cortex seed region. Reduced covariance was found overall in PTSD groups compared with non-PTSD controls between cortical thickness in the right medial Prefrontal Cortex/ Anterior Cingulate Cortex seed region of interest and cortical thickness in the left entorhinal (111 vertices, p=0.00003, corrected with RFT).

**SUPPLEMENTARY FIGURE 3**

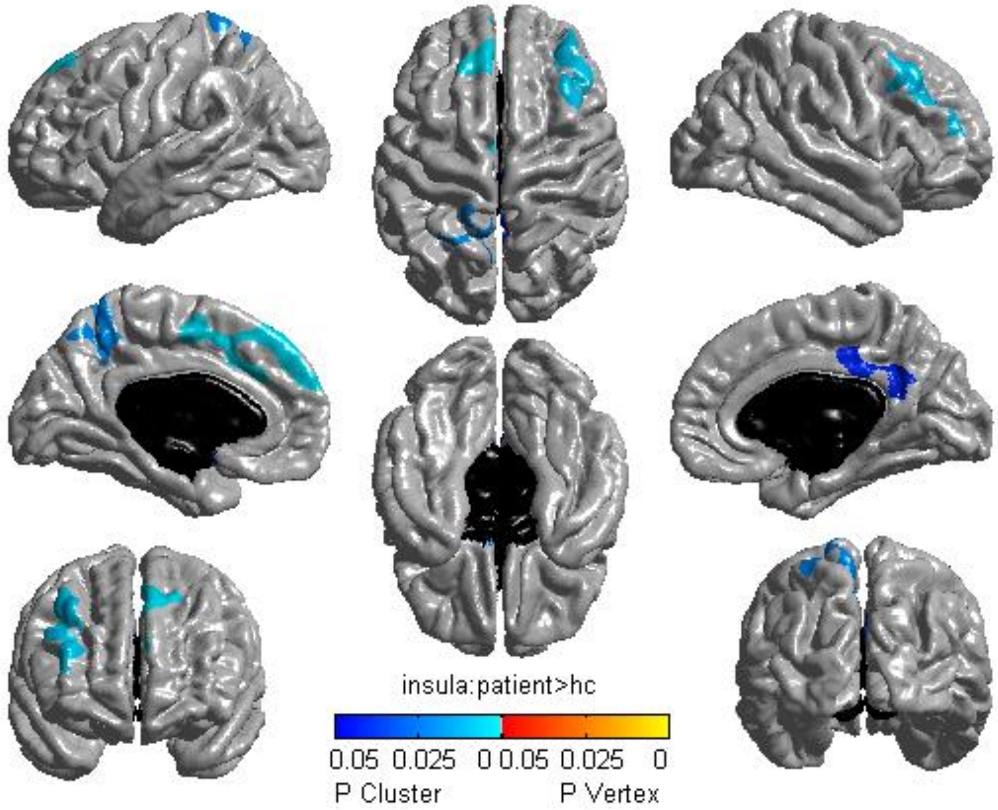


**SUPPLEMENTARY FIGURE 3** RFT-corrected map depicting seed-based structural covariance networks of the left insula seed region. Increased covariance was found overall in PTSD groups compared with non-PTSD controls between cortical thickness in the left insula seed region of interest and cortical thickness in the left superior frontal gyrus (2780 vertices, p=0.0037, corrected with RFT), right rostral middle frontal gyrus (2195 vertices, p=0.0083, corrected with RFT), left precuneus (2098 vertices, p=0.02, corrected with RFT) and right precuneus (1846 vertices, p=0.04, corrected with RFT).

**SUPPLEMENTARY FIGURE 4**

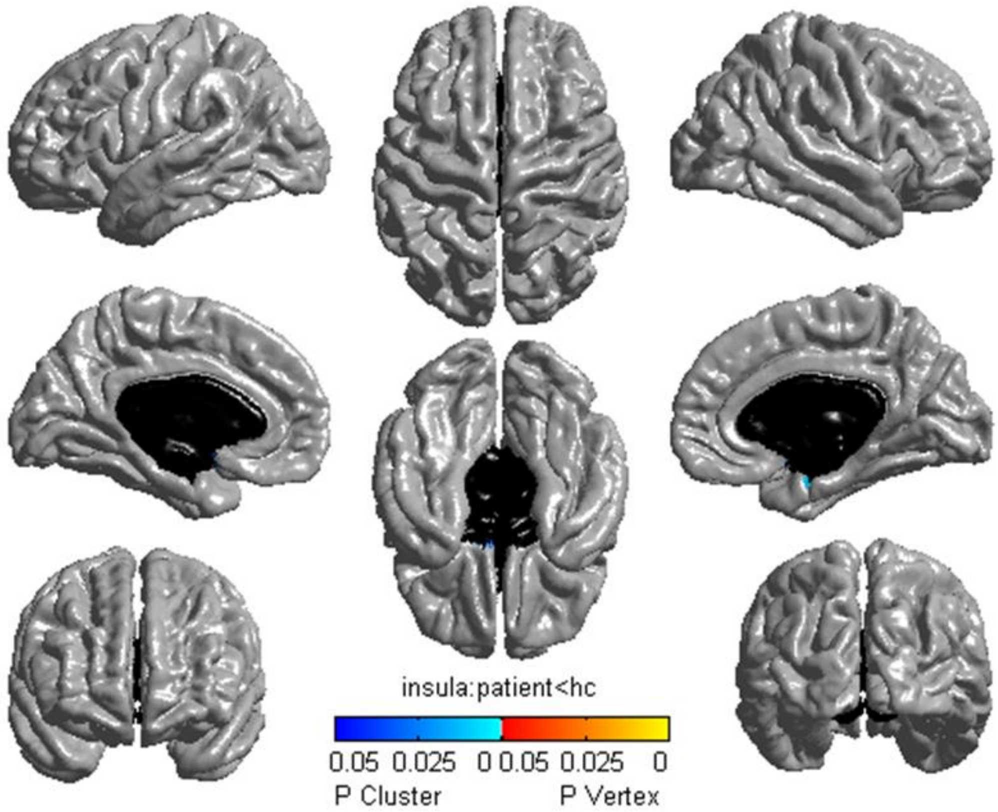


**SUPPLEMENTARY FIGURE 4** RFT-corrected map depicting seed-based structural covariance networks of the left insula seed region. Reduced covariance was found overall in PTSD groups compared with non-PTSD controls between cortical thickness in the left insula seed region of interest and cortical thickness in the right entorhinal (40 vertices, p=0.0097, corrected with RFT).

**SUPPLEMENTARY FIGURE 5**

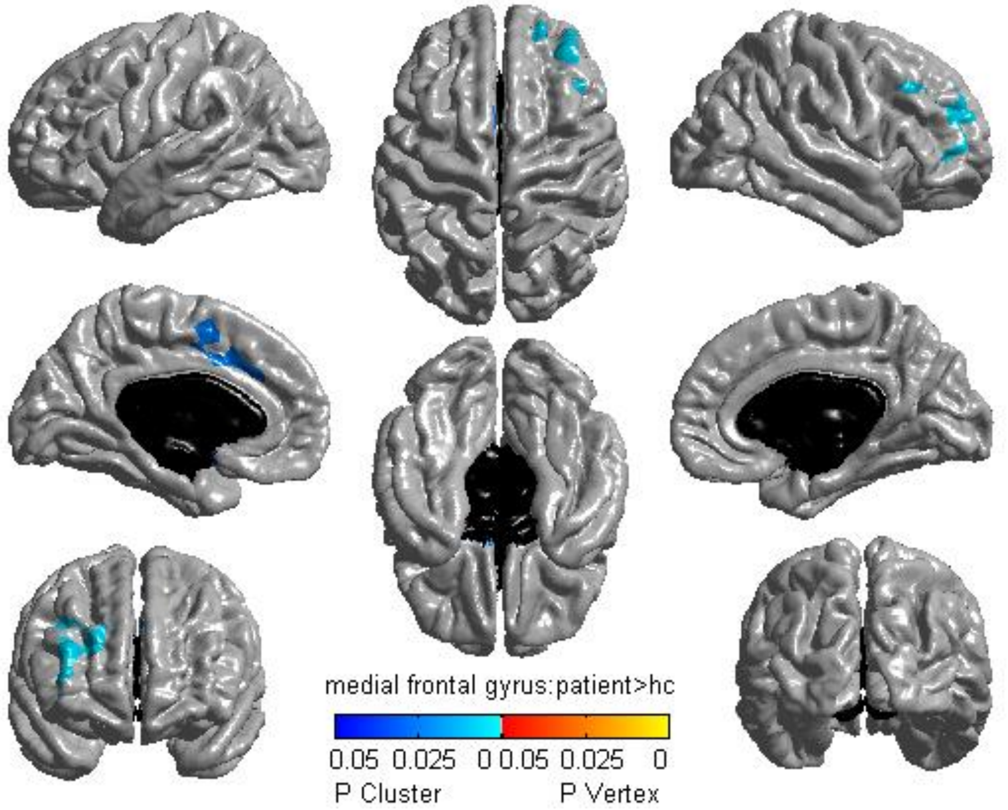


**SUPPLEMENTARY FIGURE 5** RFT-corrected map depicting seed-based structural covariance networks of the left medial frontal gyrus seed region. Increased covariance was found overall in PTSD groups compared with non-PTSD controls between cortical thickness in the left medial frontal gyrus seed region of interest and cortical thickness in the right rostral middle frontal gyrus (2029 vertices, p=0.006, corrected with RFT), left superior frontal gyrus (1767 vertices, p=0.02, corrected with RFT).

**SUPPLEMENTARY FIGURE 6**

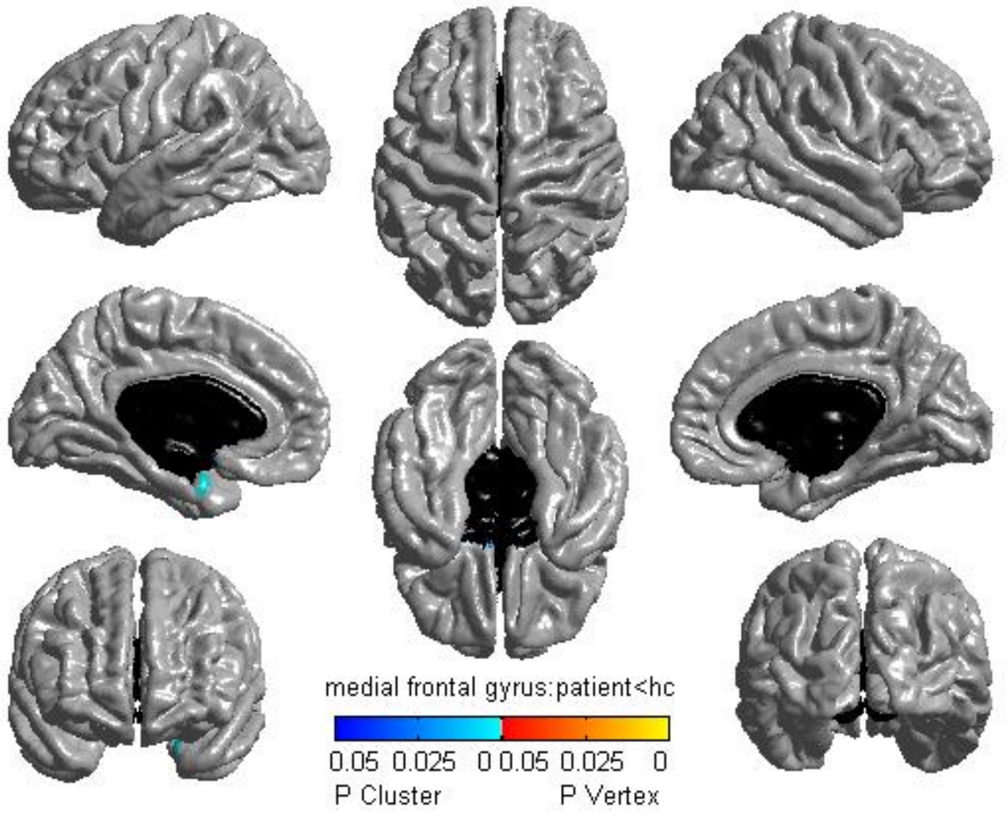


**SUPPLEMENTARY FIGURE 6** RFT-corrected map depicting seed-based structural covariance networks of the left medial frontal gyrus seed region. Reduced covariance was found overall in PTSD groups compared with non-PTSD controls between cortical thickness in the left medial frontal gyrus seed region of interest and cortical thickness in the left entorhinal (98 vertices, p=0.00003, corrected with RFT).

**SUPPLEMENTARY FIGURE 7**

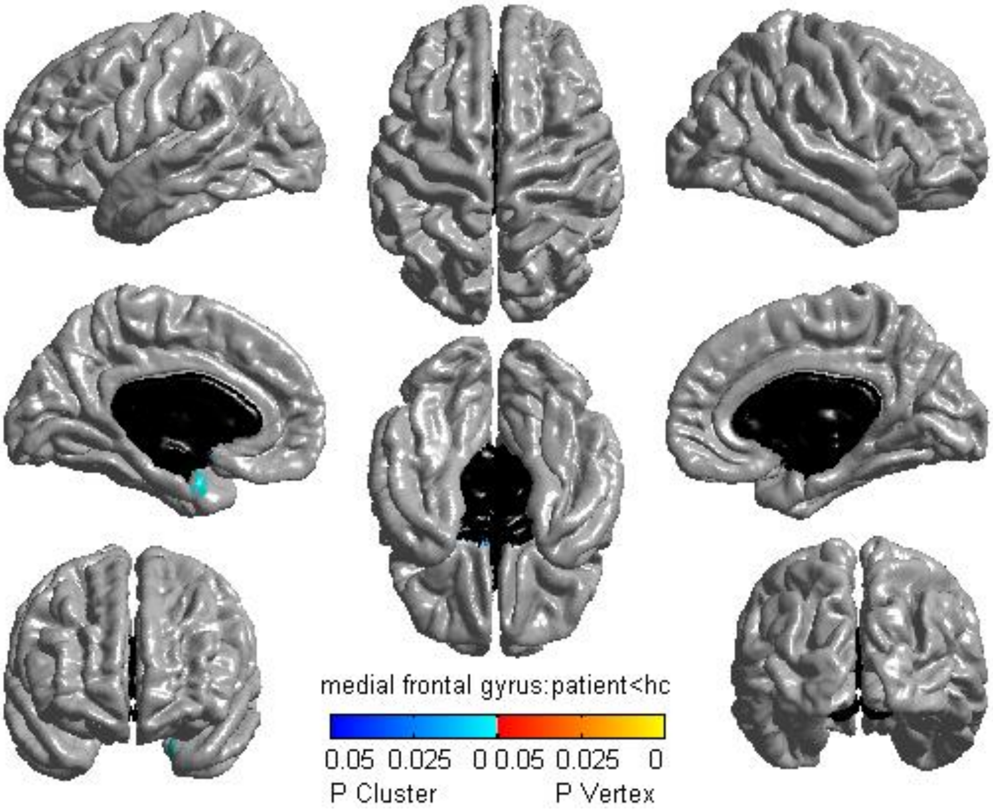


**SUPPLEMENTARY FIGURE 7** RFT-corrected map depicting seed-based structural covariance networks of the right medial frontal gyrus seed region. Reduced covariance was found overall in PTSD groups compared with non-PTSD controls between cortical thickness in the right medial frontal gyrus seed region of interest and cortical thickness in the left entorhinal (111 vertices, p=0.00004, corrected with RFT).

**SUPPLEMENTARY FIGURE 8**

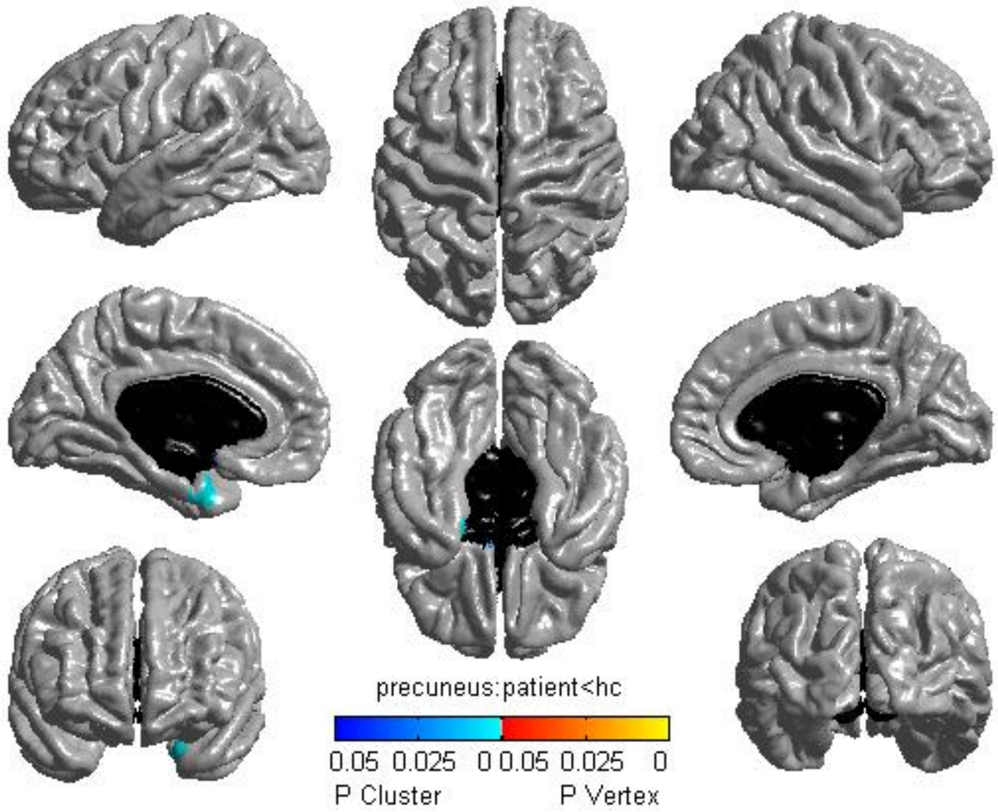


**SUPPLEMENTARY FIGURE 8** RFT-corrected map depicting seed-based structural covariance networks of the right precuneus seed region. Reduced covariance was found overall in PTSD groups compared with non-PTSD controls between cortical thickness in the right precuneus seed region of interest and cortical thickness in the left entorhinal (206 vertices, p=0.0004, corrected with RFT).

**SUPPLEMENTARY FIGURE 9**

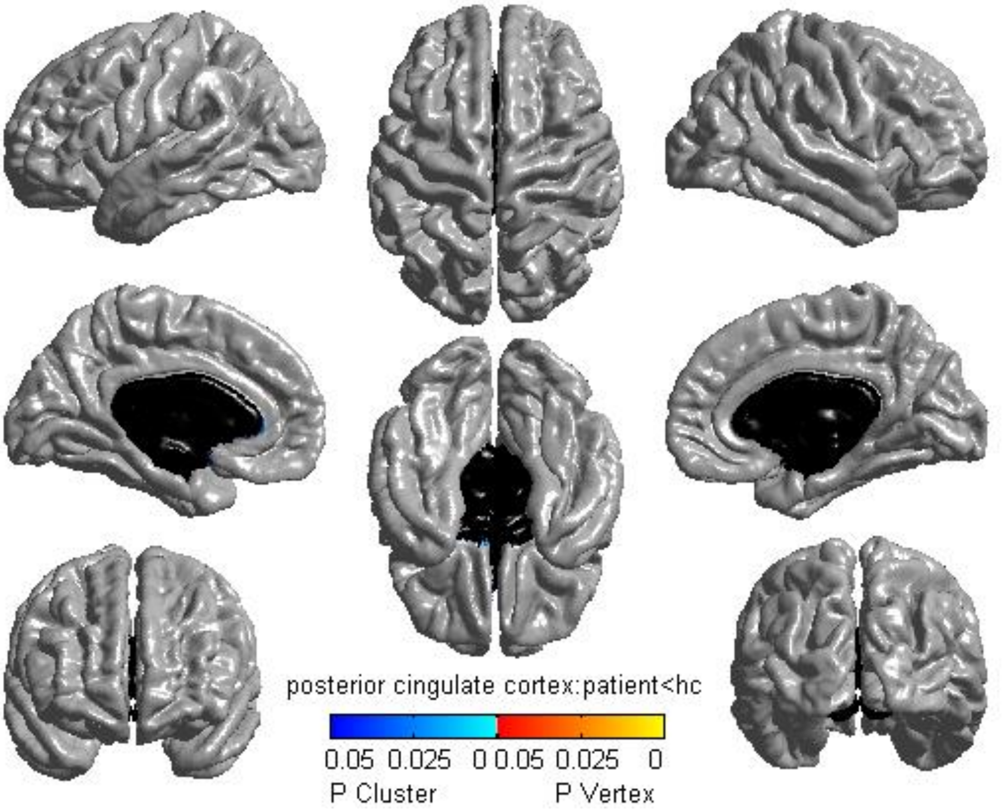


**SUPPLEMENTARY FIGURE 9** RFT-corrected map depicting seed-based structural covariance networks of the left posterior cingulate cortex seed region. Reduced covariance was found overall in PTSD groups compared with non-PTSD controls between cortical thickness in the left posterior cingulate cortex seed region of interest and cortical thickness in the left rostral anterior cingulate (74 vertices, p=0.02, corrected with RFT).

**SUPPLEMENTARY FIGURE 10**

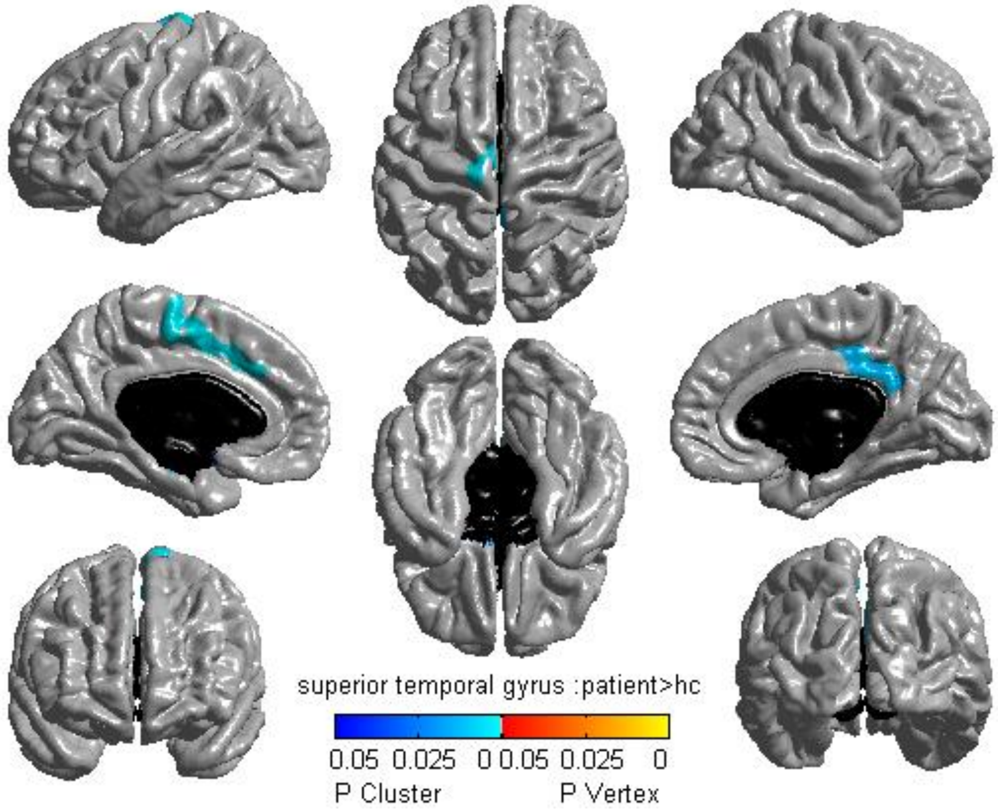


**SUPPLEMENTARY FIGURE 10** RFT-corrected map depicting seed-based structural covariance networks of the left superior temporal gyrus/angularis seed region. Increased covariance was found overall in PTSD groups compared with non-PTSD controls between cortical thickness in the left superior temporal gyrus/angularis seed region of interest and cortical thickness in the left superior frontal gyrus (2986 vertices, p=0.002, corrected with RFT), left parahippocampal (38 vertices, p=0.006, corrected with RFT), right posterior cingulate and isthmus cingulate (2204 vertices, p=0.01, corrected with RFT).

**SUPPLEMENTARY FIGURE 11**

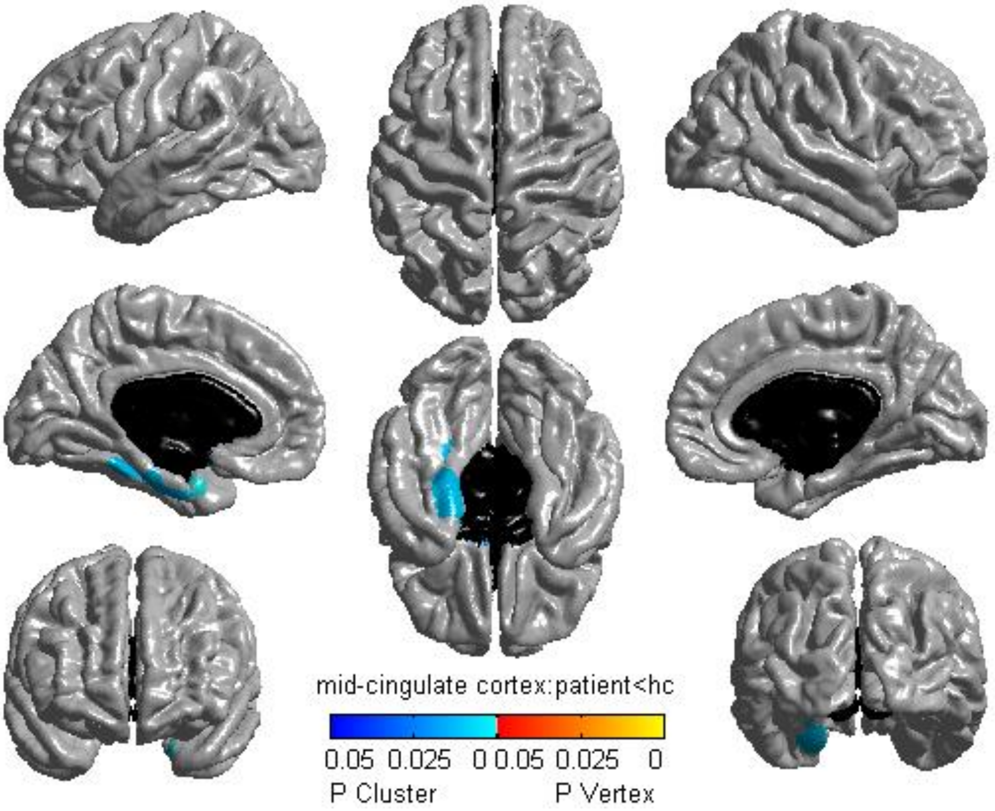


**SUPPLEMENTARY FIGURE 11** RFT-corrected map depicting seed-based structural covariance networks of the right mid-cingulate cortex seed region. Reduced covariance was found overall in PTSD groups compared with non-PTSD controls between cortical thickness in the right mid-cingulate cortex seed region of interest and cortical thickness in the left entorhinal (112 vertices, p=0.0001, corrected with RFT), left parahippocampal and entorhinal (1548 vertices, p=0.00999, corrected with RFT).

**SUPPLEMENTARY FIGURE 12**

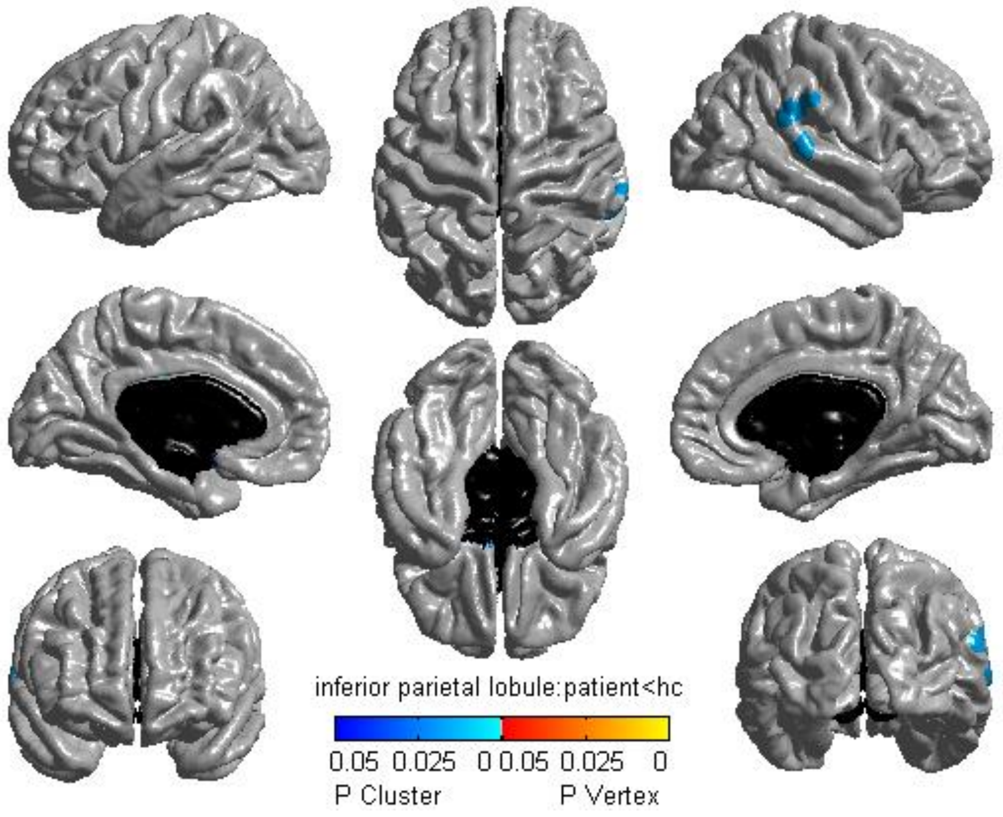


**SUPPLEMENTARY FIGURE 12** RFT-corrected map depicting seed-based structural covariance networks of the right inferior parietal lobule seed region. Reduced covariance was found overall in PTSD groups compared with non-PTSD controls between cortical thickness in the right inferior parietal lobule seed region of interest and cortical thickness in the left posterior cingulate cortex (51 vertices, p=0.0068, corrected with RFT), right supramarginal (2351 vertices, p=0.015, corrected with RFT).

**SUPPLEMENTARY FIGURE 13**

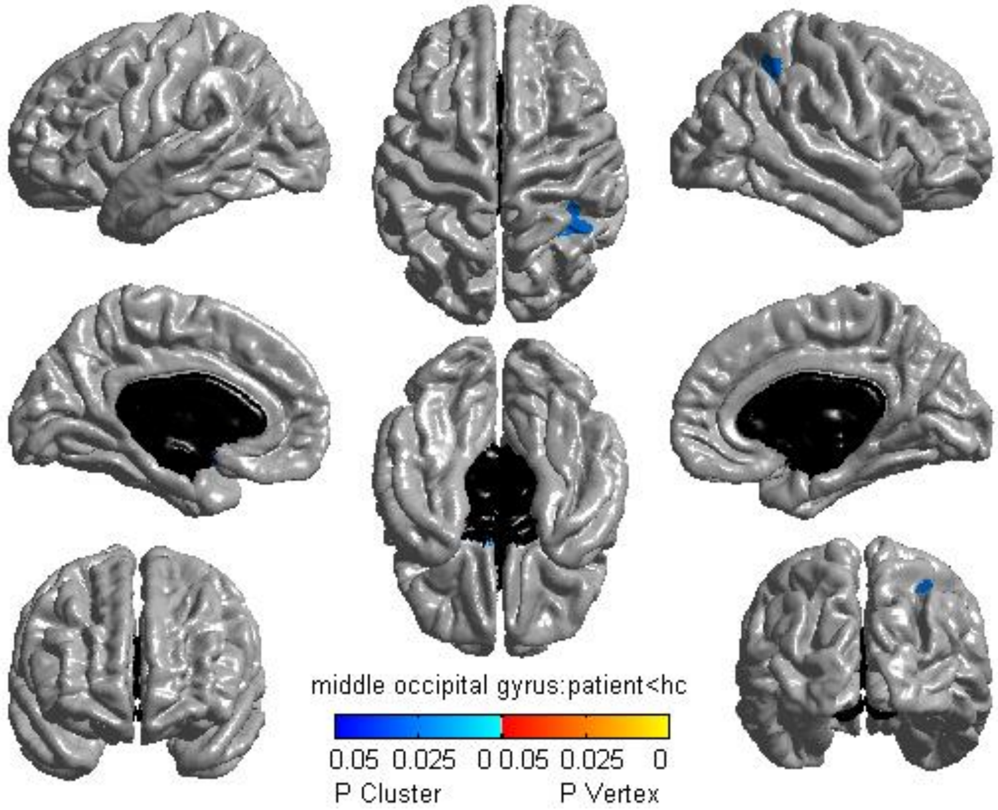


**SUPPLEMENTARY FIGURE 13** RFT-corrected map depicting seed-based structural covariance networks of the left middle occipital gyrus seed region. Reduced covariance was found overall in PTSD groups compared with non-PTSD controls between cortical thickness in the left middle occipital gyrus seed region of interest and cortical thickness in the right superior parietal gyrus (2169 vertices, p=0.02, corrected with RFT).

**SUPPLEMENTARY FIGURE 14**

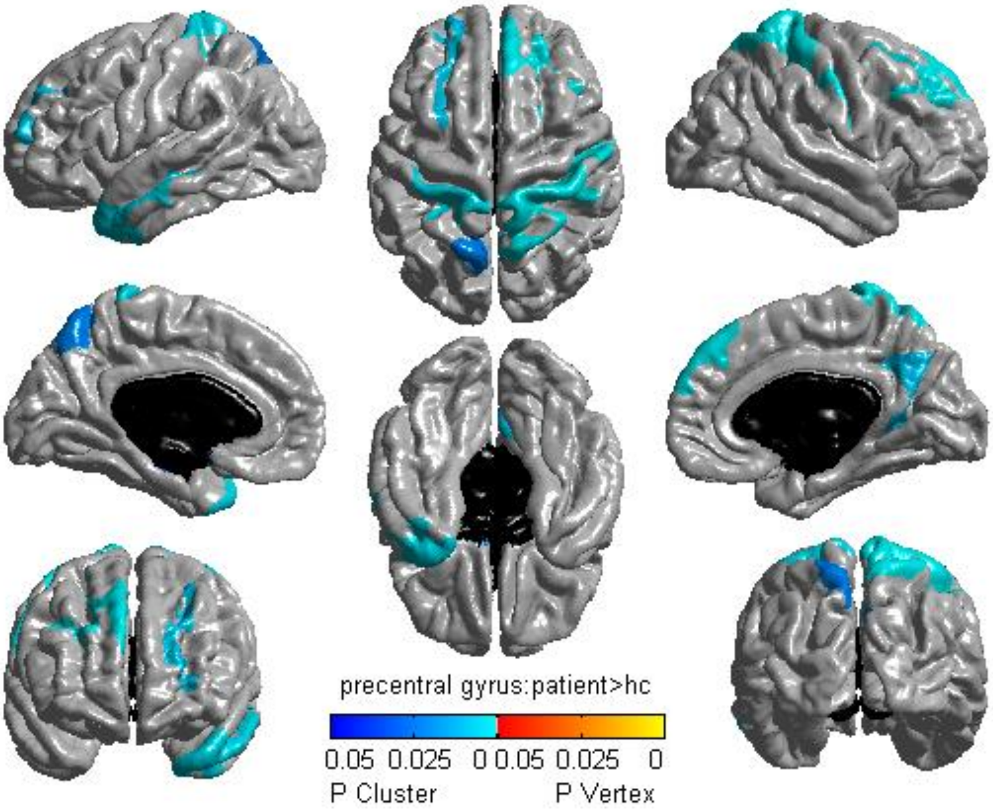


**SUPPLEMENTARY FIGURE 14** RFT-corrected map depicting seed-based structural covariance networks of the left precentral gyrus seed region. Increased covariance was found overall in PTSD groups compared with non-PTSD controls between cortical thickness in the left precentral gyrus seed region of interest and cortical thickness in the right postcentral (9343 vertices, p=0.00001, corrected with RFT), right rostral middle frontal (3613 vertices, p=0.0003, corrected with RFT), left postcentral and superior parietal (3754 vertices, p=0.003, corrected with RFT), left middle temporal and temporal pole (2701 vertices, p=0.003, corrected with RFT), left rostral middle frontal (2387 vertices, p=0.007, corrected with RFT), right precuneus and isthmus cingulate (2556 vertices, p=0.009, corrected with RFT), left superior parietal (1914 vertices, p=0.02, corrected with RFT), left parahippocampal (28 vertices, p=0.03, corrected with RFT).

**SUPPLEMENTARY FIGURE 15**

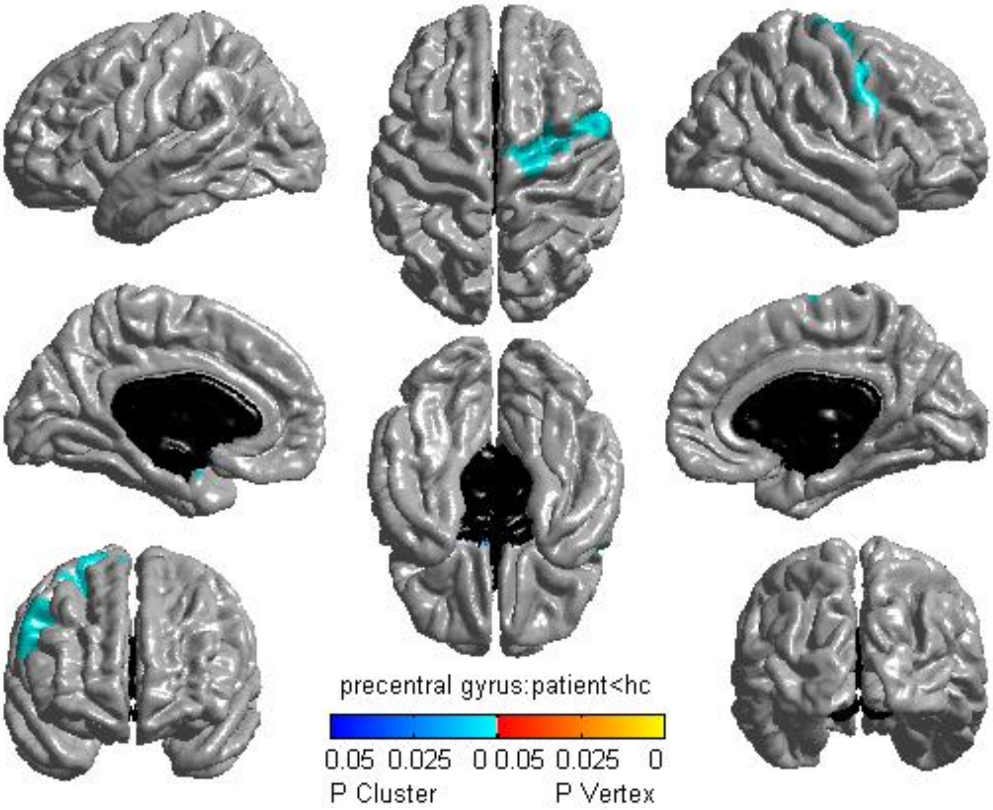


**SUPPLEMENTARY FIGURE 15** RFT-corrected map depicting seed-based structural covariance networks of the left precentral gyrus seed region. Reduced covariance was found overall in PTSD groups compared with non-PTSD controls between cortical thickness in the left precentral gyrus seed region of interest and cortical thickness in the right precentral (5375 vertices, p=0.0003, corrected with RFT), left entorhinal (54 vertices, p=0.004, corrected with RFT).

**SUPPLEMENTARY FIGURE 16**

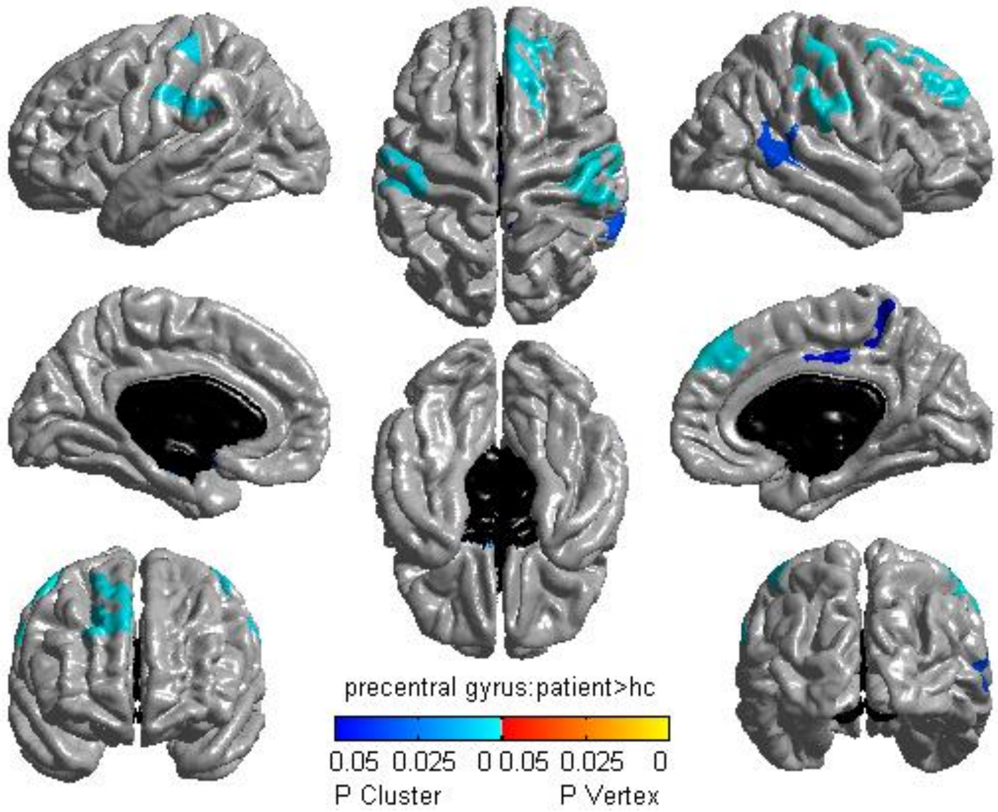


**SUPPLEMENTARY FIGURE 16** RFT-corrected map depicting seed-based structural covariance networks of the right precentral gyrus seed region. Increased covariance was found overall in PTSD groups compared with non-PTSD controls between cortical thickness in the right precentral gyrus seed region of interest and cortical thickness in the right postcentral (4605 vertices, p=0.0003, corrected with RFT), right superior frontal (3010 vertices, p=0.002, corrected with RFT), left supramarginal (3500 vertices, p=0.002, corrected with RFT), left parahippocampal (35 vertices, p=0.0089, corrected with RFT), right bankssts (2334 vertices, p=0.03, corrected with RFT), right paracentral (1731 vertices, p=0.049, corrected with RFT).

**SUPPLEMENTARY FIGURE 17**

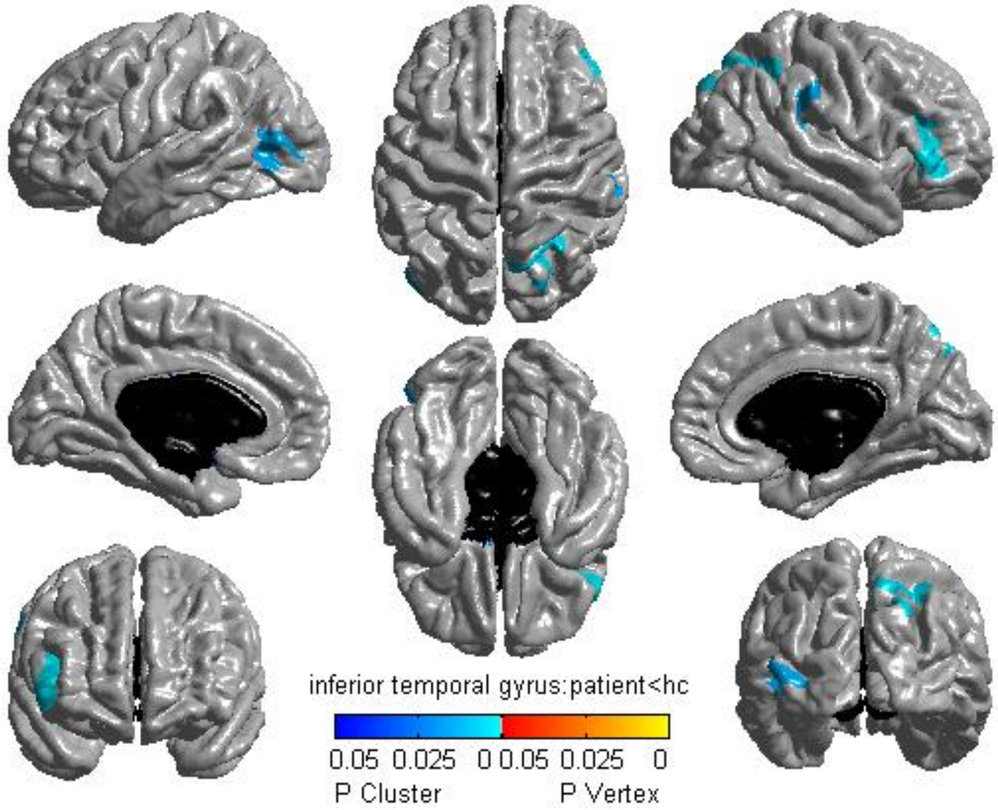


**SUPPLEMENTARY FIGURE 17** RFT-corrected map depicting seed-based structural covariance networks of the left inferior temporal gyrus seed region. Reduced covariance was found overall in PTSD groups compared with non-PTSD controls between cortical thickness in the left inferior temporal gyrus seed region of interest and cortical thickness in the right parstriangularis (2317 vertices, p=0.003, corrected with RFT), right superior parietal (2775 vertices, p=0.004, corrected with RFT), left supramarginal and superior temporal (2836 vertices, p=0.01, corrected with RFT), left lateral occipital and inferior parietal (1952 vertices, p=0.01, corrected with RFT), left posterior cingulate (48 vertices, p=0.049, corrected with RFT).

**SUPPLEMENTARY FIGURE 18**

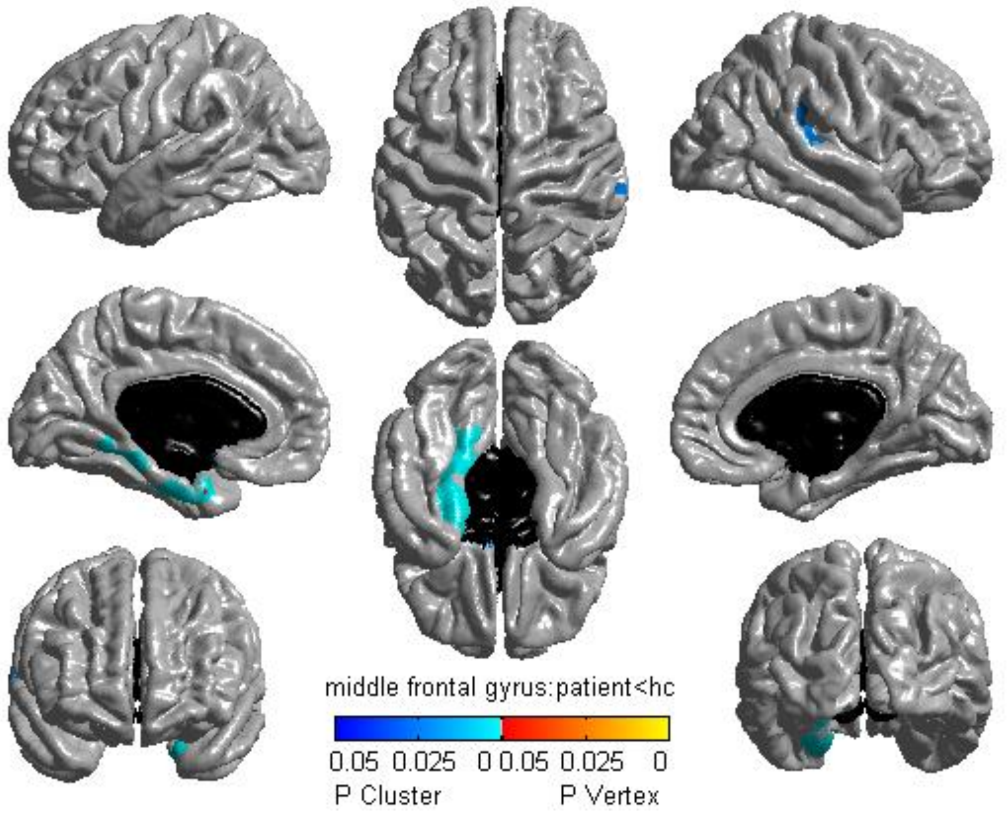


**SUPPLEMENTARY FIGURE 18** RFT-corrected map depicting seed-based structural covariance networks of the left middle frontal gyrus seed region. Reduced covariance was found overall in PTSD groups compared with non-PTSD controls between cortical thickness in the left middle frontal gyrus seed region of interest and cortical thickness in the left parahippocampal and entorhinal (2178 vertices, p=0.00002, corrected with RFT), right supramarginal and transverse temporal (2894 vertices, p=0.02, corrected with RFT).

**SUPPLEMENTARY TABLE 1**

| Category | Abbreviation | Definitions |
|---|---|---|
| ROI | HIP_L | left hippocampus |
| ROI | AMYG_L | left amygdala |
| ROI | AMYG_R | right amygdala |
| ROI | mPFC_L | left medial Prefrontal Cortex |
| ROI | mPFC_R | right medial Prefrontal Cortex |
| ROI | INS_L | left insula |
| ROI | INS_R | right insula |

| | | |
|---|---|---|
| ROI | MFG_L | left medial frontal gyrus |
| ROI | MFG_R | right medial frontal gyrus |
| ROI | CAU_L | left caudate |
| ROI | IFG_R | right inferior frontal gyrus |
| ROI | PCUN_L | left precuneus |
| ROI | PCUN_R | right precuneus |
| ROI | PCC_L | left posterior cingulate cortex |
| ROI | FFG_L | left fusiform gyrus |
| ROI | FFG_R | right fusiform gyrus |
| ROI | STG_L | left superior temporal gyrus |
| ROI | MCC_L | left mid-cingulate cortex |
| ROI | MCC_R | right mid-cingulate cortex |
| ROI | IPL_R | right inferior parietal lobule |
| ROI | MOG_L | left middle occipital gyrus |
| ROI | SFG_L | left superior frontal gyrus |
| ROI | PreCG_L | left precentral gyrus |
| ROI | PreCG_R | right precentral gyrus |
| ROI | ITG_L | left inferior temporal gyrus |
| Desikan-Killiany atlas | BANK_L | left bankssts |
| Desikan-Killiany atlas | cACC_L | left caudal anterior cingulate |
| Desikan-Killiany atlas | CMF_L | left caudal middle frontal |
| Desikan-Killiany atlas | CUN_L | left cuneus |
| Desikan-Killiany atlas | EC_L | left entorhinal cortex |
| Desikan-Killiany atlas | FFG_L | left fusiform |
| Desikan-Killiany atlas | IPG_L | left inferior parietal gyrus |
| Desikan-Killiany atlas | ITG_L | left inferior temporal gyrus |
| Desikan-Killiany atlas | ICC_L | left isthmus cingulate cortex |
| Desikan-Killiany atlas | LOC_L | left lateral occipital cortex |
| Desikan-Killiany atlas | lOFC_L | left lateral orbitofrontal cortex |
| Desikan-Killiany atlas | LING_L | left lingual cortex |
| Desikan-Killiany | mOFC_L | left medial orbitofrontal cortex |

| | | |
|---|---|---|
| atlas | | |
| Desikan-Killiany atlas | MTG_L | left middle temporal gyrus |
| Desikan-Killiany atlas | PHG_L | left parahippocampal |
| Desikan-Killiany atlas | PCL_L | left paracentral |
| Desikan-Killiany atlas | POP_L | left pars opercularis |
| Desikan-Killiany atlas | POR_L | left pars orbitalis |
| Desikan-Killiany atlas | PTRI_L | left pars triangularis |
| Desikan-Killiany atlas | PCAL_L | left pericalcarine cortex |
| Desikan-Killiany atlas | PoCG_L | left postcentral gyrus |
| Desikan-Killiany atlas | PCC_L | left posterior cingulate cortex |
| Desikan-Killiany atlas | PreCG_L | left precentral gyrus |
| Desikan-Killiany atlas | PCUN_L | left precuneus |
| Desikan-Killiany atlas | rACC_L | left rostral anterior cingulate |
| Desikan-Killiany atlas | rMFG_L | left rostral middle frontal gyrus |
| Desikan-Killiany atlas | SFG_L | left superior frontal gyrus |
| Desikan-Killiany atlas | SPG_L | left superior parietal gyrus |
| Desikan-Killiany atlas | STG_L | left superior temporal gyrus |
| Desikan-Killiany atlas | SMG_L | left supramarginal gyrus |
| Desikan-Killiany atlas | FPC_L | left frontal pole |
| Desikan-Killiany atlas | TPO_L | left temporal pole |
| Desikan-Killiany atlas | TTG_L | left transverse temporal gyrus |
| Desikan-Killiany atlas | INS_L | left insula |

| | | |
|---|---|---|
| Desikan-Killiany atlas | BANK_R | right bankssts |
| Desikan-Killiany atlas | cACC_R | right caudal anterior cingulate |
| Desikan-Killiany atlas | CMF_R | right caudal middle frontal |
| Desikan-Killiany atlas | CUN_R | right cuneus |
| Desikan-Killiany atlas | EC_R | right entorhinal cortex |
| Desikan-Killiany atlas | FFG_R | right fusiform gyrus |
| Desikan-Killiany atlas | IPG_R | right inferior parietal gyrus |
| Desikan-Killiany atlas | ITG_R | right inferior temporal gyrus |
| Desikan-Killiany atlas | ICC_R | right isthmus cingulate cortex |
| Desikan-Killiany atlas | LOC_R | right lateral occipital cortex |
| Desikan-Killiany atlas | lOFC_R | right lateral orbitofrontal cortex |
| Desikan-Killiany atlas | LING_R | right lingual cortex |
| Desikan-Killiany atlas | mOFC_R | right medial orbitofrontal cortex |
| Desikan-Killiany atlas | MTG_R | right middle temporal gyrus |
| Desikan-Killiany atlas | PHG_R | right parahippocampal |
| Desikan-Killiany atlas | PCL_R | right paracentral |
| Desikan-Killiany atlas | POP_R | right pars opercularis |
| Desikan-Killiany atlas | POR_R | right pars orbitalis |
| Desikan-Killiany atlas | PTRI_R | right pars triangularis |
| Desikan-Killiany atlas | PCAL_R | right pericalcarine |
| Desikan-Killiany atlas | PoCG_R | right postcentral gyrus |
| Desikan-Killiany atlas | PCC_R | right posterior cingulate cortex |

| | | |
|---|---|---|
| Desikan-Killiany atlas | PreCG_R | right precentral gyrus |
| Desikan-Killiany atlas | PCUN_R | right precuneus |
| Desikan-Killiany atlas | rACC_R | right rostral anterior cingulate |
| Desikan-Killiany atlas | rMFG_R | right rostral middle frontal gyrus |
| Desikan-Killiany atlas | SFG_R | right superior frontal gyrus |
| Desikan-Killiany atlas | SPG_R | right superior parietal gyrus |
| Desikan-Killiany atlas | STG_R | right superior temporal gyrus |
| Desikan-Killiany atlas | SMG_R | right supramarginal gyrus |
| Desikan-Killiany atlas | FPC_R | right frontal pole |
| Desikan-Killiany atlas | TPO_R | right temporal pole |
| Desikan-Killiany atlas | TTG_R | right transverse temporal gyrus |
| Desikan-Killiany atlas | INS_R | right insula |
